# Captive-reared monarchs tracked in the wild show southward migration: reply to Davis (2021)

**DOI:** 10.1093/conphys/coab064

**Published:** 2021-08-18

**Authors:** Alana A E Wilcox, Amy E M Newman, Nigel E Raine, Greg W Mitchell, D Ryan Norris

**Affiliations:** Department of Integrative Biology, University of Guelph, Guelph, Ontario, N1G 2W1, Canada; Department of Integrative Biology, University of Guelph, Guelph, Ontario, N1G 2W1, Canada; School of Environmental Sciences, University of Guelph, Guelph, Ontario, N1G 2W1, Canada; Wildlife Research Division, Environment and Climate Change Canada, National Wildlife Research Centre, 1125 Colonel By Drive, Ottawa, Ontario, K1S 5B6, Canada; Department of Biology, Carleton University, 1125 Colonel By Drive, Ottawa, Ontario, K1S 5B6, Canada; Department of Integrative Biology, University of Guelph, Guelph, Ontario, N1G 2W1, Canada

We thank Davis (2021) for their comments on our recent paper in *Conservation Physiology* (Wilcox *et al.,* 2021), where we report the results of an experiment showing that captive-reared monarch butterflies (*Danaus plexippus*) released in the wild with radio-transmitters were able to orient southwards during fall migration. In contrast, monarchs reared under the same conditions but flown in a flight simulator did not show a strong tendency towards southward migration. Davis (2021) raised three main concerns related to (i) the orientation of monarch butterflies during fall migration, (ii) the lack of a control group and (iii) the potential negative impact of the devices used in both orientation tests. We address each of these issues below.

## Orientation of monarch butterflies during fall migration

Davis (2021) points out that, while that evidence suggests the eastern North American population of monarch butterflies follows a southwest trajectory during fall migration (Mouritsen and Frost, 2002; Mouritsen *et al.,* 2013; Parlin *et al.,* 2021), radio-tracked monarchs in Wilcox *et al.* (2021) principally flew southeast to south. While southwest may be the primary direction reported in some studies, there is substantial individual variation in flight direction in migratory monarch butterflies. Mouritsen *et al.* (2013) conducted flight simulation tests on 23 wild-caught migratory monarch butterflies in southwestern Ontario during the fall and, while mean direction from this sample was 215° (southwest), 39% of individuals had a mean flight direction between 90° (east) and 180° (south). Given individual differences in monarch orientation, it is entirely feasible that studies with smaller sample sizes, such as ours, could have a population circular mean that deviates from the expected south to southwest orientation.

It is also possible that a southeast direction actually reflects the true migratory direction of monarchs in southern Ontario, which is geographically unique as monarchs in SW Ontario must navigate the Great Lakes. Knight *et al.* (2019) released 43 wild-caught monarchs tagged with miniaturized radio-transmitters and obtained 21 unique detections from 12 individual monarchs at automated radio receiving towers south of the capture site, resulting in a population mean direction of 172° (southeast). While this was also a small sample size, we now have two studies—one from wild-caught monarchs and another from captive-reared monarchs—that have shown fall migratory monarchs released in southern Ontario show an average southeast direction (with substantial variation). As such, if latitudinal and/or longitudinal variation in flight direction exists, we caution against directly comparing orientation from monarchs in southern Ontario to those measured in Cincinnati, OH (Parlin *et al.,* 2021), and Ithaca, NY (Schmidt-Koenig, 1985). Future studies examining regional variation in orientation with latitude or geographical context are ultimately needed. Regardless of the factors driving deviation from the south to southwest orientation, there was a clear difference between the southward orientation of radio-tracked monarchs and the lack of directional flight observed in monarchs flown in our flight simulator (Wilcox *et al.,* 2021).

## Lack of control group

Davis (2021) expressed concern that a control group of wild-caught monarch butterflies was not radio tracked at the same time as captive-reared monarchs. This is a fair point and something we already addressed in Wilcox *et al.* (2021). Captive-reared radio-tracked monarchs in our study principally flew southeast to south and, while we were unable to compare directly to a wild-caught control group, these results are consistent with the orientation of wild-caught monarch butterflies captured during the fall in the same region (Knight *et al.,* 2019). Monarchs tested in the flight simulator showed no strong orientation in any direction, which contrasts markedly with wild-caught monarchs tested in the same area (Mouritsen *et al.,* 2013) and our captive-reared monarchs tracked in the wild (Knight *et al.,* 2019; Wilcox *et al.,* 2021). So, while there was no wild-caught control group tested at the same time as captive-reared monarchs, there is no reason to expect that this would vary over different years, making us confident in our results.

## Tracking considerations

We agree with Davis (2021) that the use of radio transmitters may cause deleterious effects on animals and that unexpected behavioural responses during radio tracking are most often attributed to the weight and attachment of devices (Hagen *et al.,* 2011; Balmori, 2016). The tags in our study have been successfully used in prior studies on monarchs (Knight *et al.,* 2019) and were modified to the smallest size possible. Monarchs in our study were >300 mg at release, and with the additional weight of a small radio-transmitter tag the total mass was still within the normal range for this species (i.e. 300–750 mg; Brindza *et al.,* 2008; Pocius *et al.,* 2017). Nonetheless, we agree that the distribution of weight on the monarch may not perfectly reflect the normal fat distribution and encourage further investigation into the effect of radio-tracking tags on monarch butterflies.

The procedures for attaching the radio transmitters and the tungsten rod to tether monarchs in the flight simulator could impact monarch flight. However, all efforts were made to ensure that monarchs had a full range of motion and showed characteristic patterns of flight (i.e. strong flapping with intermittent gliding). Though it is possible that methods used to secure the tungsten rod could result in reduced survival (Parlin *et al.,* 2021), we also note that monarchs have survived multi-week experiments with tethers secured in their thorax. Mouritsen *et al.* (2013) conducted flight simulation tests up to 5 times per day on monarch butterflies in Guelph, Ontario, then drove individuals with the tungsten rod still attached 3000 km to Calgary, Alberta, where they were flown again in the flight simulator 8–21 days after the initial test. Therefore, we suggest that this method likely has little short-term effect on the ability of monarchs to display migratory orientation.

Experimental approaches offer a unique opportunity to gain insight into the mechanisms driving variation in migration success, yet few studies have combined laboratory and field techniques (Birnie-Gauvin *et al.,* 2020). Our study reports findings on the impact of captive rearing under specific controlled environmental conditions on monarch butterfly flight orientation in both laboratory and field. As we discuss in Wilcox *et al.* (2021), if captive rearing were used as a tool for conservation, efforts must be made to not only minimize risks, but also determine the optimal rearing and environmental conditions for successful migration. This discussion brings to light some of the challenges in studying migratory species and we encourage the research community to thoroughly examine the strengths and limitations of captive rearing on monarch butterflies, as well as continue to work on finding solutions to mitigate population declines.
